# Biomarkers of Type IV Collagen Turnover Reflect Disease Activity in Patients with Early-Stage Non-Alcoholic Fatty Liver (NAFL)

**DOI:** 10.3390/biology12081087

**Published:** 2023-08-04

**Authors:** Ida Lønsmann, Jane I. Grove, Asma Haider, Philip Kaye, Morten A. Karsdal, Diana J. Leeming, Guruprasad P. Aithal

**Affiliations:** 1 Nordic Bioscience Biomarkers and Research A/S, 2730 Herlev, Denmark; 2 Department of Clinical Research, Faculty of Health Sciences, University of Southern Denmark, 5000 Odense, Denmark; 3 NIHR Nottingham Biomedical Research Centre, Nottingham University Hospitals NHS Trust, University of Nottingham, Nottingham NG7 2UH, UK; 4 MRC/EPSRC Nottingham Molecular Pathology Node, University of Nottingham, Nottingham NG7 2UH, UK; 5 Nottingham Digestive Diseases Centre, Translational Medical Sciences, School of Medicine, University of Nottingham, Nottingham NG7 2UH, UK; 6 Department of Pathology, Nottingham University Hospitals NHS Trust, Nottingham NG7 2UH, UK

**Keywords:** biomarkers, basement membrane, NAFLD, extracellular matrix, collagen turnover

## Abstract

**Simple Summary:**

The Global increase in obesity and type II diabetes has led to a rapid increase in liver disease caused by fat accumulation in the liver. Therefore, it is of utmost importance to find blood-based markers that may enable the identification of persons with early-stage disease, especially since new interventions are being developed within this area. We found that blood-based biomarkers of a highly specialized tissue, known as the basement membrane, may relate to early-stage disease in patients that have fatty-liver involvement without inflammation. Such markers may in the future aid in finding early-stage steatosis; however, this needs to be further investigated.

**Abstract:**

Background: Identification of progressive liver disease necessitates the finding of novel non-invasive methods to identify and monitor patients in need of early intervention. Investigating patients with early-liver injury may help identify unique biomarkers. Early-liver injury is characterized by remodeling of the hepatocyte basement membrane (BM) of the extracellular matrix. Thus, we quantified biomarkers targeting two distinct neo-epitopes of the major BM collagen, type IV collagen (PRO-C4 and C4M), in patients spanning the non-alcoholic fatty liver disease (NAFLD) spectrum. Methods: We evaluated PRO-C4 and C4M in a cross-sectional study with 97 patients with NAFLD confirmed on histology. Serological levels of PRO-C4 and C4M were quantified using validated competitive enzyme-linked immunosorbent assays (ELISA). Using the fatty liver inhibition of progression (FLIP) algorithm, we stratified patients into two groups: non-alcoholic fatty liver (NAFL) and non-alcoholic steatohepatitis (NASH). Biomarker levels were investigated in the two groups in patients stratified by the NAFLD activity score (NAS). In both groups, biomarker measurements were analyzed in relation to histological scorings of steatosis, inflammation, ballooning, and fibrosis. Results: Patients had a body mass index (BMI) of 30.9 ± 5.6 kg/m^2^, age of 53 ± 13 years and a NAS range of 1–8. Upon stratification by FLIP, the NASH patients had higher platelets, ALT, and AST levels than the NAFL group. Both PRO-C4 (*p* = 0.0125) and C4M (*p* = 0.003) increased with increasing NAS solely within the NAFL group; however, a large variability was present in the NASH group. Furthermore, both markers were significantly associated with lobular inflammation (*p* = 0.020 and *p* = 0.048) and steatosis (*p* = 0.004 and *p* = 0.015) in patients with NAFL. Conclusions: This study found that type IV collagen turnover increased with the increase in NAS in patients with NAFL; however, this was not the case in patients with NASH. These findings support the assessments of the BM turnover using biomarkers in patients with early-disease development. These biomarkers may be used to track specific processes involved in the early pathobiology of NAFL.

## 1. Introduction

Advancing therapies of liver diseases may in the future expand from focusing on end-stage liver disease to reversing or preventing disease progression in patients with early-stage liver disease including screening patients in the general population [1]. To accomplish this, biomarkers that accurately describe early-disease activity are needed. Markers might be used alone to monitor early-stage patients or in combination with already established biomarkers in the field, such as PRO-C3 a marker of active fibrogenesis, or the two composite scores “A PRO-C3-based fibrosis algorithm that included age, presence of diabetes, PRO-C3, and platelet count” (ADAPT) or the “Enhanced Liver Fibrosis score” (ELF), developed for the detection of advanced liver fibrosis in patients with liver disease, that are best suited for the identification and monitoring of late-stage liver fibrosis, F3-F4 [2,3].

Non-alcoholic steatohepatitis (NASH) is an important entity in the spectrum of non-alcohol fatty liver disease (NAFLD) to identify due to its potential in the development of fibrosis which is the best predictor of NAFLD-related mortality [4,5]. Increasing stages of fibrosis are characterised by the formation of bridging septa mainly composed of fibrillar type I and III collagens in the interstitial space of the extracellular matrix [6,7]. In contrast, lower stages of fibrosis in NAFLD are associated with pericellular fibrosis in the sinusoids and have been proposed to be formed by basement-membrane components, like laminin and type IV collagen [6,7,8]. Compared to other organs, the basement membrane found in a healthy liver is somewhat less dense [9]. However, upon injury, the pericellular basement membrane is formed [8]. Thus, it may be possible to measure turnover of the basement membrane as a novel tool to non-invasively detect disease activity at an early stage.

The aim of this preliminary study was to investigate the association of type IV collagen neoepitope biomarkers with histological characteristics of the NAFLD activity score in patients with NAFL in comparison to patients with NASH. Thus, we explored the relevance of assessing basement-membrane turnover in liver disease pathophysiology at different stages of disease progression.

## 2. Methods

### 2.1. Study Participants

A total of 97 biopsy-proven patients with an NAFLD activity score (NAS) of 1–8 were enrolled. The study was performed according to the ethical guidelines of the Declaration of Helsinki. Patients gave informed written consent for recruitment to one of the following studies: GM010201 approved by the East Midlands Nottingham 2 NRES Committee; 12/WM/0288 approved by the West Midlands NRES Committee; or 09/H0403/1 approved by the Nottingham NHS Ethics Committee. The study participants were prospectively recruited consecutively from hepatology clinics at the Nottingham University Hospitals NHS Trust between 2009 and 2018. NAFLD was diagnosed on the basis of the following criteria: a liver biopsy showing histology consistent with NAFLD; a weekly ethanol consumption of <14 units for women and <21 units for men; appropriate exclusion of other causes of liver disease including alcohol, drugs, autoimmune or viral hepatitis, or cholestatic or metabolic/genetic liver disease.

### 2.2. Histological Assessement

Demographic data were recorded, and blood samples were taken at the study visit within 3 months of undergoing a diagnostic liver biopsy. Serum was prepared and stored at −80 °C within 4 h. Histological parameters (steatosis, inflammation, hepatocyte ballooning and fibrosis) were scored using the Clinical Research Network (CRN) system [10]. The grade of NASH was assessed by an experienced histopathologist using the NAFLD activity score (NAS), scored from 0–8, incorporating scores of steatosis (0–3), ballooning (0–2), and lobular inflammation (0–3). Following recruitment, 4 patients were excluded as suitable samples were not available for analysis.

### 2.3. Scores Applied in This Study

According to the fatty liver inhibition of progression (FLIP) algorithm/SAF score, for each biopsy, an SAF score summarizing the main histological lesions was defined. This assesses both the grade of steatosis (S), and separately, the grade of activity (A), and the stage of fibrosis (F), the latter according to the NASH Clinical Research Network (CRN) [11]. The enhanced liver fibrosis panel (ELF) consist of a composite score including tissue inhibitor of metalloproteinases 1 (TIMP-1), N-terminal propeptide of type III collagen (PIIINP) and hyaluronic acid (HA) [12]. The ADAPT score [13], a composite of a neoepitope marker of N-terminal propeptide of type IIII collagen, PRO-C3, presence of type II diabetes, platelets and fatty liver index were all used. The fatty liver index (FLI) consists of waist circumference, serum triglycerides, BMI and gamma-glutamyl transpeptidase (GGT) [14].

### 2.4. Basement-Membrane-Turnover Biomarkers

The biomarkers reflecting basement-membrane remodelling of collagen type IV, PRO-C4 [15] and C4M [16] were assessed as previously described using competitive ELISAs developed by Nordic Bioscience. For both markers, streptavidin-coated 96-well plates were incubated with biotinylated antigen coater reflecting the targeted peptide sequence of each of the biomarkers. Twenty µL of serum samples, controls and standards were added to appropriate wells and incubated with horseradish peroxidase-labelled monoclonal antibodies as specified for each assay. Finally, optical density was measured at 450 nm with 650 nm as reference and the biomarker level was calculated from the standard curve by the SoftMax Pro v.7.0.3 software (Molecular Devices, San Jose, CA, USA). All incubation steps were performed at 300 rpm followed by five washes of the plates (25 mM Trizma-base, 50 mM NaCl, 0.1% Tween20, pH 7.2).

### 2.5. Statistics

Patients were split into two groups based on FLIP score defined as (1) NAFLD, with steatosis and ballooning or inflammation, or (2) NASH, with the presence of both steatosis, ballooning, and inflammation. Differences in continuous clinical characteristics between the two groups were compared using one-way ANOVA or the Kruskal–Wallis test where appropriate. Categorical variables were compared using Fisher’s exact test. Levels of the type IV collagen biomarkers were analysed in relation to NAS in both the NAFL and NASH groups, as well as sub scores of hepatocyte ballooning, lobular inflammation, steatosis and fibrosis. Differences in PRO-C4 and C4M were analysed using either the Mann–Whitney U test or Kruskal–Wallis one-way analysis of variance where appropriate. *p*-values < 0.05 were considered significant.

## 3. Results

### 3.1. Patient Characteristics

The total study population consisted of 97 NAFLD patients with proven steatosis, as presented in Table 1. The patients had a mean BMI of 31 kg/m^2^, age of 53 years and ranged from NAS 1–8. Among them, 65 patients had NASH with presence of hepatocyte ballooning and lobular inflammation in addition to steatosis. The remaining 32 patients were classified as NAFL according to the SAF score. When comparing the two groups, fasting glucose (*p* = 0.022), triglycerides (*p* = 0.047), ALT (*p* = 0.045), and AST (*p* = 0.005) levels were significantly elevated in patients with NASH compared to NAFL, while platelets (*p* = 0.038) and HDL (*p* = 0.035) were significantly lower. Selected multivariable biomarker composite scores were also elevated in patients with NASH patients compared to patients with NAFL: the enhanced liver fibrosis panel (ELF) [12] (*p* = 0.002), ADAPT [13] (*p* < 0.001), and the fatty liver index (FLI) [14] (*p* = 0.038). In addition, patients in the NASH group had a higher degree of liver fibrosis compared to NAFL (*p* = 0.003). Lastly, there was no difference in in PRO-C4 and C4M between the NAFL and NASH groups.

### 3.2. Basement-Membrane Turnover Is Associated with NAS in NAFL, but Not within NASH

**Figure 1:**
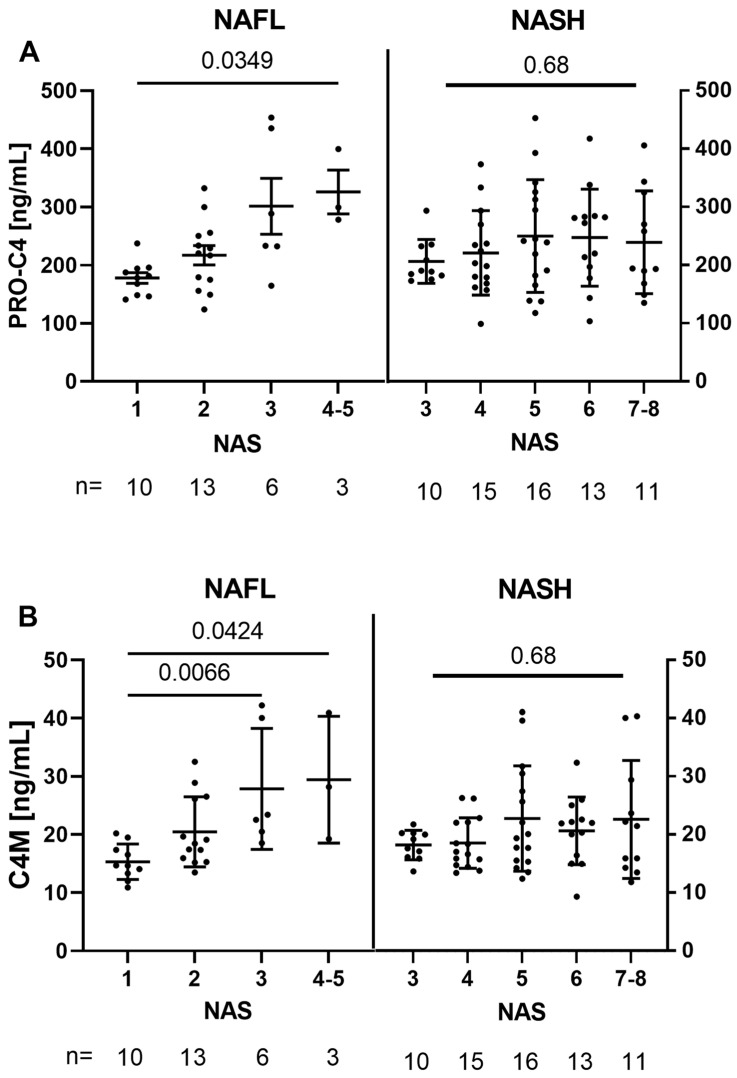
Graphs showing measurements of the neo-epitopes PRO-C4 (**A**), and C4M (**B**) stratified by the NAS score. Abbreviations: NAFL: non-alcoholic fatty liver; NAS: non-alcoholic fatty liver disease activity score; NASH: non-alcoholic steatohepatitis.

Serum levels of the BM biomarkers were comparable between the NASH and NAFL subgroups. When stratified based on the NAFLD activity score (NAS) in the two subgroups, as shown in Figure 1, interestingly, both PRO-C4 and C4M increased with NAS in the NAFL subgroup (*p* = 0.0125 and *p* = 0.0030). However, this tendency did not apply for the NASH group. Within the NAFL subgroup, C4M showed higher levels in patients with NAS 3 compared to NAS 1 (*p* = 0.0066). Furthermore, there was a difference between NAS 4–5 and NAS 1 (*p* = 0.0304 for PRO-C4 and *p* = 0.0424 for C4M). However, only three patients were classified as NAS 4–5.

### 3.3. BM Biomarkers Reflect Histological Features of Early Liver Damage in Patients with NAFL

**Figure 2:**
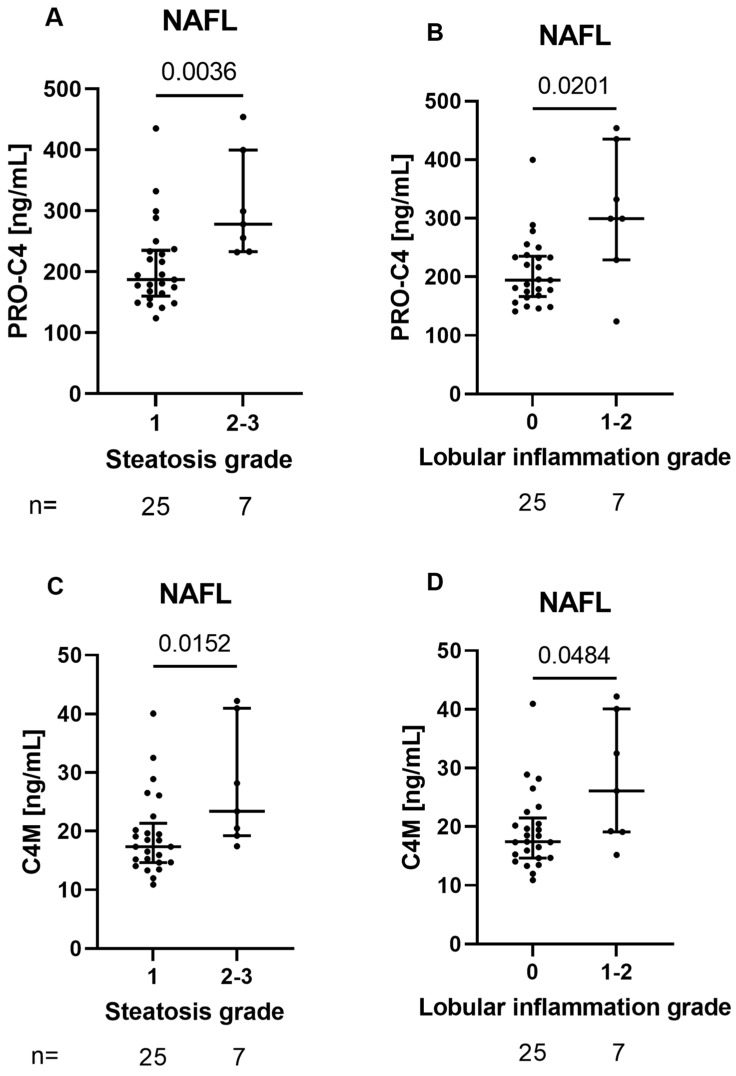
The top-row graphs show serum levels of the neo-epitope PRO-C4 stratified by histological gradings of steatosis (**A**) and lobular inflammation (**B**). Lower-row graphs show serum levels of the neo-epitope C4M stratified by histological gradings of steatosis (**C**) and lobular inflammation (**D**). Abbreviations: NAFL: non-alcoholic fatty liver.

Based on the correlation between the BM biomarkers, PRO-C4 and C4M, and NAS in NAFL patients, we investigated the association between the histological components of NAS hepatocyte ballooning, lobular inflammation, and steatosis. As seen in Figure 2, Both PRO-C4 and C4M were significantly elevated in patients with lobular inflammation (stage 1–2) compared to no inflammation (stage 0) (*p* = 0.020 and *p* = 0.048, respectively). Additionally, PRO-C4 and C4M were elevated in patients with medium-to-high steatosis (stage 2–3) compared to a low degree of steatosis (stage 1) (*p* = 0.004 and *p* = 0.015, respectively). There were no significant associations between the serum BM biomarkers and fibrosis stage or hepatocyte ballooning. Additionally, no associations between the serum BM biomarkers and histological scorings were found for patients with NASH. 

## 4. Discussion

The results presented in this article show two important things: (1) as hypothesised by various researchers, basement-membrane remodelling may be detected in patients with signs of early liver damage and (2) that this remodelling is indeed associated with liver histopathology in early-stage liver disease.

The formation of a basement membrane with the presence of type IV collagen in the liver sinusoids is not a new concept. However, while most evaluations of type IV collagen have been shown in animals [17,18], the presence of type IV collagen has also been shown by liver immunohistochemistry in human liver tissue originating from various aetiologies [7,19,20]. In a study, utilizing tissue from liver biopsy from patients with alcoholic liver disease (ALD) or hepatitis C, collagen type IV seems to be located within both the sinusoidal fibrosis and the fibrotic bands depending on stage. However, type IV collagen has been shown to be present in early-stage disease, but located in the perisinusoidal space [19]. The investigations in the current study propose that changes in type IV collagen may be assessed in blood samples which correlate to the histopathology of patients with NAFL, and not in patients with NASH.

Recently, it has been stated that there is a need for non-invasive tests for both the assessment of liver-fibrosis quantity as well as disease activity. By histology, activity has generally been defined as hepatocyte ballooning and lobular inflammation [21]. Both these features, especially, ballooning, are nevertheless very difficult to assessed with low inter- and intra-observer variation [22]. Within the current study, we showed an association between increasing levels of BM remodelling as assessed by PRO-C4 and C4M with increasing scores of histologically assessed lobular inflammation and steatosis. This agrees with a previous study showing that high levels of PRO-C4 correlated to steatosis and lobular inflammation independently of fibrosis stage in a study that investigated remodelling of collagens in alcohol-related liver disease [23]. However, a correlation to hepatocyte ballooning was also observed in that study [23]. In the current study, no correlation was observed between hepatocyte ballooning and biomarkers of type IV collagen remodelling in patients with NAFL or NASH. However, it should be noted that upon stratification into histological sub scores, the number of patients in each group was low. Ultimately, as lobular inflammation often precedes fibrosis in the progression of liver disease [24], type IV collagen might reflect these early stages of liver pathophysiology and might have utility as an early marker of disease activity.

It can be observed that PRO-C4 and C4M becomes more variable in patients with NASH compared to NAFL; thus, overall, it looks like BM remodelling does not relate to NAS. Both markers assessed are consistent in this aspect. Nevertheless, the variation may be due to different individual responses of BM-remodelling activity in patients with NASH. It may also be that since NASH is a metabolic-driven disease, it may affect other organs such as the gut in which there is a large amount of BM. We cannot exclude that PRO-C4 and C4M may also be reflecting gut involvement in NASH. From the data, it appears that a non-invasive measure of BM remodelling may be used in NAFL but not in NASH.

As this exploratory study is limited by a small number of patients upon stratification into each of the NAS sub scores and lacks a validation cohort, further testing is required before the clinical use of these biomarkers can be fully evaluated.

## 5. Conclusions

Remodelling of the basement membrane type IV collagen positively correlated to severity of hepatic steatosis and lobular inflammation in NAFL patients, but not NASH patients. These biomarkers may be used to track specific processes involved in the early pathobiology of NAFL only.

## Figures and Tables

**Table 1:** Patient characteristics of the 97 NAFLD patients studied. Patients were divided into either NAFL or NASH based on histological scorings of liver biopsies. The *p*-value reports difference between the NAFL and NASH subgroups.

PARAMETER	ALL (N = 97)	NAFL (N = 32)	NASH (N = 65)	*p*-VALUE
**BMI, KG/M^2^**	30.9 (±5.6)	29.58 (±5.76)	31.48 (±5.36)	0.125
**AGE, YEARS**	53 (±13)	50 (±16)	54 (±11)	0.181
**SEX, FEMALE**	38.1%	34.4%	40%	0.754
**DIABETES, YES**	41.2%	28.1%	47.7%	0.081
**PLATELETS, 10^9^/L**	229.0 (185–283)	254 (202.5–298)	217 (178–268)	**0.038**
**BILIRUBIN,** **µ** **MOL/L**	10.0 (8.0–13.0)	10.0 (7.0–13.0)	11.0 (9.0–14.0)	0.263
**ALBUMIN, G/L**	39.0 (38.0–42.0)	38.0 (37.0–41.0)	40.0 (38.0–43.0)	0.102
**ALT, U/L**	56.0 (38.0–81.0)	47.5 (30.5–69.0)	57.0 (39.0–92.0)	**0.045**
**AST, U/L**	39.0 (30.0–55.0)	31.5 (25.75–37.5)	40.0 (34.0–71.0)	**0.005**
**GGT, U/L**	85.0 (57.2–165.0)	96.5 (48.5–150.75)	84.0 (60.0–169.25)	0.729
**FERRITIN,** **µ** **G/L**	168.0 (66.2–308.2)	221 (97.5–306.5)	152 (61.5–321.5)	0.766
**FASTING GLUCOSE, MMOL/L**	5.3 (4.7–6.8)	4.9 (4.5–6.0)	5.5 (4.9–7.2)	**0.022**
**HDL, MMOL/L**	1.3 (1.0–1.6)	1.4 (1.1–1.8)	1.1 (1.0–1.4)	**0.035**
**LDL, MMOL/L**	2.7 (1.8–3.5)	3.0 (2.1–3.3)	2.7 (1.6–3.6)	0.358
**TRIGLYCERIDES, MMOL/L**	1.8 (1.2–2.9)	1.7 (1.1–2.2)	2.2 (1.4–3.1)	**0.047**
**ELF**	9.2 (8.5–10.1)	8.6 (7.9–9.6)	9.3 (8.8–10.4)	**0.002**
**ADAPT**	6.7 (5.3–9.0)	5.3 (4.5–6.8)	7.6 (5.9–9.5)	**<0.001**
**FLI**	84.4 (70.3–94.2)	77.8 (65.7–84.4)	88.6 (73.1–96.7)	**0.038**
**NAFLD ACTIVITY SCORE, N**				
**1/2/3/4/5/6/7/8**	10/13/16/17/17/13/9/2	10/13/6/2/1/0/0/0	0/0/10/15/16/13/9/2	**<0.001**
**STEATOSIS, N**				
**1/2/3**	47/24/26	25/3/4	22/21/22	**<0.001**
**LOBULAR INFLAMMATION, N**				
**0/1/2/3**	47/24/26	25/6/1	0/41/19	**<0.001**
**HEPATOCYTE BALLOONING, N**				
**0/1/2**	19/37/41	19/10/3	0/27/38	**<0.001**
**FIBROSIS, N**				
**0/1/2/3/4**	24/15/18/27/12	14/7/5/3/2	10/8/13/24/10	**0.003**
**PRO-C4, NG/ML**	219 (178–282)	219 (173–261)	219 (179–283)	0.645
**C4M, NG/ML**	19 (16–23)	19 (15–24)	19 (16–23)	0.933

Abbreviations: BMI: body mass index; ALT: alanine aminotransferase; AST: aspartate aminotransferase; GGT: gamma-glutamyltransferase; HDL: high-density lipoprotein; LDL: low-density lipoprotien; ELF: enhanced liver fibrosis score; ADAPT: a PRO-C3-based fibrosis algorithm that included age, presence of diabetes, PRO-C3, and platelet count; FLI: fatty liver index; NAFLD: non-alcohol fatty liver disease; PRO-C4: type IV collagen formation; C4M: Type IV collagen degradation. Bold *p*-values: Statistically significant p-values.

## Data Availability

No new data were created or analysed in this study. Data sharing is not applicable to this article due to GDPR.
